# CRLX101 (formerly IT-101)–A Novel Nanopharmaceutical of Camptothecin in Clinical Development

**DOI:** 10.2174/157340711795163866

**Published:** 2011-03

**Authors:** Cissy Young, Thomas Schluep, Jungyeon Hwang, Scott Eliasof

**Affiliations:** 1 Cerulean Pharma Inc., 840 Memorial Drive, Cambridge MA 02139, USA; 2 Independent consultant to Cerulean Pharma Inc., USA

**Keywords:** Nanopharmaceutical, oncology, camptothecin, topoisomerase 1.

## Abstract

CRLX101 (formerly IT-101) is a first-in-class nanopharmaceutical, currently in Phase 2a development, which has been developed by covalently conjugating camptothecin (CPT) to a linear, cyclodextrin-polyethylene glycol (CD-PEG) co-polymer that self-assembles into nanoparticles. As a nanometer-scale drug carrier system, the cyclodextrin polymeric nanoparticle technology, referred to as “CDP”, has unique design features and capabilities. Specifically, CRLX101 preclinical and clinical data confirm that CDP can address not only solubility, formulation, toxicity, and pharmacokinetic challenges associated with administration of CPT, but more importantly, can impart unique biological properties that enhance CPT pharmacodynamics and efficacy.

## 1 INTRODUCTION AND RATIONALE

Polymeric nanoparticles are promising drug carriers. Research and development with drug-containing nanoparticles has shown that they can enhance aqueous solubility, prolong plasma half-life, and reduce systemic toxicity of chemotherapeutic agents *in vitro* and *in vivo *[1,2]. However, more recent findings with polymeric nanoparticles have illustrated their unique ability to introduce additional biological advantages compared to other drug delivery technologies and approaches, such as liposomes, polymeric micelles, and prodrug conjugates.

Polymeric nanoparticles as drug carriers can be designed to have defined size and surface properties to favor drug deposition and retention in tumors by extravasation through disorganized leaky vasculature commonly found in tumors and inflamed tissues, described as the enhanced permeability and retention (EPR) effect [3,4]. The nanoparticle’s physical attributes can also be optimized to facilitate target tissue penetration and cellular uptake, thereby overcoming drug resistance by circumventing surface-pump mediated multi-drug resistance (e.g. P-glycoprotein-mediated) mechanisms [2]. Furthermore, the physical and chemical properties can be designed to incorporate stealth properties and maintain physical integrity, which can shield drug molecules from rapid systemic clearance, degradation, and metabolism while in circulation, and prevent broad systemic drug dissemination and exposure in healthy tissues and organs. Finally, polymeric nanoparticles can be engineered to incorporate drugs by covalent conjugation, which can provide control over drug-release kinetics that could have a significant impact on pharmacokinetics and a meaningful improvement in the drug’s pharmacodynamics and efficacy [2,5].

**Fig. (1):**
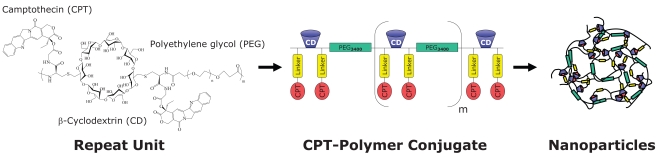
Schematic diagram of CRLX101, a nanopharmceutical comprised of camptothecin conjugated to a linear, cyclodextrin-polyethylene glycol (CD-PEG) co-polymer and formulated into nanoparticles.

CRLX101 is a polymeric nanoparticle pharmaceutical, developed with the proprietary cyclodextrin polymeric nanoparticle (CDP) technology [6,7]. It is comprised of camptothecin (CPT) covalently conjugated to a linear, cyclodextrin-polyethylene glycol (CD-PEG) co-polymer, which self-assembles into nanoparticles (Fig. **1**). Originally designed to address formulation challenges associated with CPT, a highly insoluble and labile small molecule, CRLX101 is a nanopharmaceutical that has proven to augment CPT efficacy by (a) facilitating target tissue localization and retention, (b) increasing intracellular CPT deposition, (c) providing a sustained supply of active CPT, (d) maintaining intracellular CPT concentrations above therapeutic threshold, and (e) prolonging activity at the CPT target. 

### 1.1 Background

The 20S-isomer of CPT, a natural alkaloid isolated from the Chinese bush *Camptotheca acuminata*, was first identified in the National Cancer Institute (NCI) Anticancer Drug Screen as a promising cancer drug candidate with significant anti-tumor activity [8]. Its mechanism of action was later discovered to be potent inhibition of topoisomerase 1 (Topo 1), which is an important and validated drug target for cancer therapy today. Topo 1 remains a highly attractive drug target because it is essential for basic cellular processes including DNA replication, recombination, and transcription, which are particularly up-regulated in rapidly dividing tumor cells [9]. A significant medicinal chemistry effort has been devoted to developing CPT analogs. There are currently two FDA-approved small molecule CPT analogs, topotecan (Hycamtin^®^, GlaxoSmithKline) and irinotecan (Camptosar^®^, Pfizer) [10]. While they are highly potent like CPT, both drugs exhibit sub-optimal pharmacokinetics and have significant dose-limiting toxicities, including bone marrow suppression and gastrointestinal disorders, respectively. As such, a better tolerated CPT molecule with enhanced efficacy and improved pharmacokinetics is a highly attractive cancer drug candidate that could have a significant and favorable clinical impact. 

Given its original promise as an anti-tumor agent, CPT was advanced into the clinic soon after its discovery. However, development of CPT as a drug molecule was halted because of its challenging physicochemical and pharmaceutical properties. Firstly, CPT is highly water-insoluble (approximately 4 μg/mL) and is therefore not amenable to traditional pharmaceutical formulations. Secondly, the chemical structure of CPT includes an unstable lactone ring that is highly susceptible to spontaneous and reversible hydrolysis, which yields an inactive, but more water-soluble, carboxylate form that predominates at physiologic pH [11,12]. This inactive carboxylate form was also found to bind human serum albumin with 200-fold greater affinity than the active lactone form, therefore perpetuating its predominance in circulation upon parenteral administration [13]. Clinical formulation development effort at that time did not take into consideration the need to lock CPT in its active lactone form and to protect it from rapid hydrolysis upon administration. In fact, early clinical studies were conducted with the inactive carboxylate form of CPT, which resulted in observations of significant drug-related toxicity but limited anti-tumor efficacy [14,15]. Any further advancement of CPT as a drug molecule will require formulations that release CPT in its active lactone form and maintain its biological activity by protecting it from hydrolysis.

From a mechanistic standpoint, a CPT therapeutic effect is anticipated to be maximized with improved pharmacokinetics; that is, lengthening its half-life and prolonging the bioavailability of the active lactone form. The rationale for an improved pharmacokinetic profile is two-fold. Firstly, CPT works by binding and stabilizing the transient DNA-Topo 1 cleavable complex in a reversible manner. A short drug exposure or periodic drug withdrawal would likely allow cells to evade cell death. Sustained CPT exposure and prolonged inhibition of Topo 1 in cells undergoing active DNA synthesis is therefore expected to enhance CPT pharmacodynamics and resulting efficacy [16]. Additionally, recent work at the NCI suggests that prolonged Topo 1 inhibition can mediate a novel Topo 1-dependent hypoxia-inducible factor 1 alpha (HIF-1α) inhibition mechanism [17], another promising cancer drug target that has been the subject of intense research and screening efforts. In these studies, the researchers found that maximal HIF-1α inhibition and enhanced anti-tumor efficacy is best achieved with Topo 1 inhibitors possessing extended pharmacokinetics, or with a daily low-dose dosing regimen to achieve a higher overall drug level over a longer period of time [18]. Together, these findings suggest that extending the pharmacokinetics of a Topo 1 inhibitor can prolong its on-target effect as well as augment its efficacy, potentially by activating a novel dual-inhibition mechanism.

### 1.2 Design of CRLX101

**Fig. (2):**
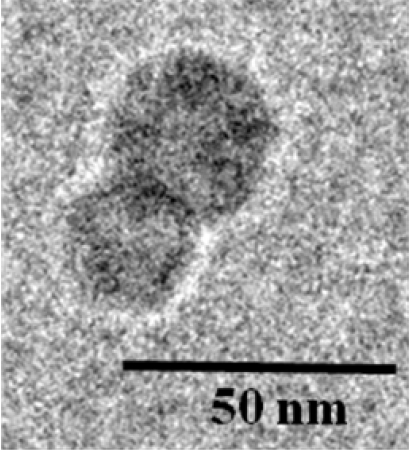
Cryo-TEM of CRLX101.

CRLX101 was designed to maximize CPT efficacy by addressing its formulation and pharmacokinetic hurdles. The chemical structure of CRLX101 is comprised of a linear co-polymer backbone incorporating alternating repeat units of cyclodextrin (CD) and polyethylene glycol (PEG) blocks with intervening chemical linkers for CPT conjugation. One of the key design features of CDP is that the CD blocks form inclusion complexes with hydrophobic small molecule drugs, such as CPT, through both intra- and inter-molecular interactions. Such interactions between adjacent polymer strands are essential for catalyzing the self-assembly of several CD-PEG polymer strands into highly reproducible nanoparticles with diameters between 30 and 40 nm [19] (Fig. **2**). The resulting drug-containing nanoparticles have neutral surface charge and the PEG blocks impart improved solubility and stealth properties, which together enable safe systemic administration, minimize immunogenicity, and help evade phagocytic uptake by the reticuloendothelial system [20]. As a result, CRLX101 exhibits extended plasma stability with prolonged circulation time, avoids rapid renal and systemic clearance, and accumulates in target tissues through the EPR effect. 

Two other major design features of CDP as a drug carrier are the covalent linkage of CPT to the CD-PEG polymer and the physical integrity of the resulting self-assembled nanoparticles. To form CRLX101, CPT is derivatized at the 20-OH position with the natural amino acid glycine to form an ester linkage for covalent attachment to CD-PEG. *In vitro* chemical characterization studies confirmed that this linker strategy successfully stabilizes the labile lactone ring of CPT in its closed, active form and prevents premature CPT inactivation by pH-mediated ring opening upon systemic administration [6]. In addition, the physical integrity of CRLX101 nanoparticles helps protect CPT from metabolic enzymes and premature hydrolysis. Consequently, only the active form of CPT is released in a controlled manner and over a sustained period of time.

Biocompatibility is another important design feature of CDP as a drug carrier technology. Preclinical animal study data demonstrated that the CD-PEG polymer, in the absence of conjugated drug, is well tolerated, eliciting no observable side effects or immune responses, despite dosing up to 240 mg/kg in mice [6]. In CRLX101, the intra- and inter-molecular interactions of CD-PEG polymer strands that catalyze inclusion complex formation are lost upon CPT release, resulting in the disassembly of the nanoparticles into individual polymer strands with hydrodynamic diameters of < 10 nm. Such individual polymer strands are then sufficiently small and inert for clearance through the kidneys. In essence, CRLX101 has been designed as a CPT depot that disassembles into biocompatible components that can be safely excreted. These preclinical findings are consistent with our clinical experience of patients tolerating multiple cycles of CRLX101 therapy without experiencing any unusual or unexpected non-drug related toxicity caused by the presence of CD-PEG co-polymer.

In summary, CDP is a highly versatile nanopharmaceutical technology that has several design features that address challenging pharmaceutical hurdles, as exemplified by its ability to substantially improve CPT solubility, maintain CPT biological activity upon administration, and control sustained CPT release, resulting in improved tolerability and enhanced efficacy *in vivo *[6,7].

## 2 PRECLINICAL CHARACTERIZATION OF CRLX101

### 2.1 *In Vitro* Studies with CRLX101

To demonstrate the controlled and sustained CPT release from CDP, the drug release kinetics and mechanism of CRLX101 were studied extensively *in vitro*. Cleavage of the glycine linker was found to be mediated through both enzymatic and base-catalyzed hydrolysis of the ester bond, with observed half-lives of 59 hours and 41^1^ hours in PBS and human plasma, respectively. While CPT release kinetics were observed to be relatively accelerated in plasma compared to in PBS, the rate of drug release is slower than what is typically observed with polymeric pro-drugs with an ester linkage [21]. It should also be noted that the *in vivo* terminal half-life in rat, dog, and human are similar (34.6 hours, 28.8 hours, and 31.8 hours, respectively), suggesting that hydrolysis of the glycine linker is not rate-limiting. In addition, the slow and sustained CPT release from CRLX101 also illustrates the physical shielding effects of CDP forming intact nanoparticles, which protect the CPT payload and minimize the enzymatic and chemical hydrolytic degradation. 

Another key characteristic of CRLX101 as a nanopharmaceutical is its ability to enter cells and release the CPT payload intracellularly. To assess the degree of cellular uptake and intracellular localization, cell culture studies were conducted by incubating fluorescently-labeled CDP-based nanoparticles with exponentially growing PC-3 cells. In these studies, CDP nanoparticles were observed to co-localize with the dye Lysotracker^®^, indicating that cellular uptake of intact nanoparticles is mediated through an endosomal pathway [7]. Since the release kinetics of CPT from CRLX101 are slow under acidic conditions typically found in these compartments, exposure of cancer cells to CRLX101 is therefore anticipated to accumulate in endosomal and lysosomal compartments where CPT release is catalyzed by intravesicular esterases. As a result, the active form of CPT is released in a slow, sustained manner from CRLX101 from an intracellular CPT depot. In a 48-hour cytotoxicity assay, the IC_50_ values for CRLX101 were observed to be in the sub-micromolar range in a variety of human cancer cell lines (Table **1**). The 2 to 10 times lower *in vitro* potency of CRLX101 compared CPT is consistent with the relatively slow CPT release from CRLX101 and with the data from the *in vitro* release studies described above. 

### 2.2 *In Vivo* Studies with CRLX101

A key goal for the design of CRLX101 is to enhance drug deposition in tumor tissues in order to increase efficacy and improve systemic tolerability. To assess localized drug concentrations in target tissues, the biodistribution of CRLX101 was evaluated in xenograft tumor-bearing mice (Table **2**). In these studies, tumor samples were harvested from animals that were dosed with either CRLX101 or CPT, and assessed for localized drug concentrations. Remarkably, in tumor tissues harvested from animals treated with CRLX101 at its maximum tolerated dose (MTD), significantly higher intratumoral concentrations of released CPT were measured at 24 and 48 hours post-administration, compared to tumor CPT concentrations found in animals treated with CPT at its MTD. These observations strongly support CRLX101 as a nanopharmaceutical that can effectively enhance CPT localization and retention in tumor tissues by the EPR effect. Furthermore, a gradual increase in released CPT level in tumor relative to plasma was observed, increasing from approximately a 2.5:1 tumor-to-plasma ratio at 24 hours to 21:1 at 48 hours post-administration. The observed increase in unconjugated CPT in tumor relative to plasma over a 24-hour period suggests that CPT may be released from CRLX101 localized within the tumor by the EPR effect, in addition to CPT distributing from plasma to tumor. These observations are in sharp contrast with the data observed in animals administered with CPT (Table **2**).

Drug concentrations in plasma and tumor were further assessed in tumor-bearing mice comparing CRLX101 with another Topo 1 inhibitor, irinotecan, a small-molecule prodrug of another active Topo 1 inhibitor, SN-38 [22]. In these experiments, drug levels were assessed in plasma samples and tumor biopsies harvested after administration of irinotecan or CRLX101 at their respective MTDs. The results showed that, at 24 and 48 hours post-administration, plasma and tumor concentrations of both CRLX101 and released CPT were greater than four orders of magnitude and greater than two orders of magnitude higher than those of irinotecan and SN-38, respectively. These findings are correlative with the significantly enhanced efficacy observed with CRLX101 compared to irinotecan in these xenograft tumor models. Most remarkably, the enhanced efficacy and increased tumor drug concentrations were correlated with increased inhibition of Topo 1 enzymatic activity in two of the tumor models (Daudi and Karpas 299) at 48 hours post-administration [22]. In summary, findings from the CRLX101 biodistribution studies provide substantive evidence that CDP nanopharmaceuticals can increase the efficacy of the active molecule by enhancing and sustaining higher localized drug concentrations in target tissues.

Another mechanism by which CPT levels are high in tumors but low in the bloodstream is the physical integrity of CRLX101 as a nanopharmaceutical. The self-assembly of CRLX101 into intact nanoparticles keeps the CPT cargo intact, prevents premature CPT release or metabolism in circulation, maximizes tumor localization through the EPR effect, and minimizes systemic drug dissemination. To explore the extent that CRLX101 stays intact in circulation, multi-organ pharmacokinetics were assessed in tumor-bearing mice in a PET/CT-study using ^64^Cu-labeled CRLX101 [19]. Results from these experiments showed that there was a high tumor accumulation of CRLX101 as intact nanoparticles, likely by extravasation through the leaky vasculature in tumor tissue. This finding was further supported by confocal immunofluorescence microscopy that visualized intact nanoparticles of CRLX101 inside tumor cells 24 hours after administration. These are compelling results illustrating that the CDP technology can significantly modulate the pharmacokinetics, and consequently the pharmacodynamics, of CPT to enhance its efficacy with prolonged drug exposure [16], i.e. keeping CPT in its active form, increasing tumor drug concentrations, augmenting intracellular drug concentration, and maintaining drug supply in target cells over longer periods of time to sustain Topo 1 and possibly HIF-1α inhibition.

One of the basic goals for the design of CRLX101 is to improve the pharmacokinetics of CPT, increasing its circulation time and improving the bioavailability of the active lactone form of CPT. CRLX101 pharmacokinetics were assessed in Sprague Dawley^®^ rats. In these studies, plasma samples were drawn from animals that were administered with either CRLX101 or CPT, and analyzed for CPT drug levels as either released drug or nanoparticle-bound drug. The mean pharmacokinetic parameters are summarized in Table **3**. In summary, relative to the parent drug CPT, pharmacokinetics of CRLX101 had a larger area under the curve, lower volume of distribution, longer terminal half-life, and slower systemic clearance [23]. In animals that were dosed with CRLX101, plasma concentrations of released CPT stayed well below those of nanoparticle-bound CPT by a factor of >30-fold at all time points, illustrating that CRLX101 is an effective drug depot, providing a slow, continuous drug supply while remaining in circulation and accumulating in target tissues.

To further explore the relationship between the improved pharmacokinetics and enhanced pharmacodynamics of CRLX101, tolerability and efficacy were studied with varying dosing schedules in rodents (Table **4**). Tolerability of CRLX101 was assessed in athymic nude mice and was observed to have a strong schedule-dependency. Dosing schedules with administration frequencies in excess of once per week were shown to be toxic, requiring significant dose reductions. This is likely due to high drug accumulation from multi-dose regimens, given CRLX101’s long terminal half-life and slow sustained CPT drug release kinetics. At lower dose frequencies, however, CRLX101 was well tolerated at dose levels up to 18 mg/kg. Efficacy of CRLX101 with a variety of dosing schedules was evaluated in nude mice bearing subcutaneous human LS174T colorectal cancers and compared to irinotecan [24]. At all dosing schedules, CRLX101 was found to be superior to CPT or irinotecan dosed at their respective MTDs^2^. Notably, weekly dosing was found to be highly efficacious and just as effective as that achieved with more frequent dosing. The weekly dosing schedule was found to be better tolerated, which is consistent with the reported released CPT pharmacokinetics and biodistribution.

A comprehensive evaluation of CRLX101 anti-tumor activity has also been completed in eleven different subcutaneous human cancer models, including LS174T and HT29 colorectal cancer, A2780 and SK-OV 3 ovarian cancer, H1299 non small-cell lung cancer, H69 small-cell lung cancer, Panc-1 pancreatic cancer, MDA-MB-231 breast cancer, along with four disseminated xenograft models, including TC71-luc Ewing’s sarcoma, Daudi, Karpas 299, and L540 lymphomas [22,24]. In every case, a single treatment cycle of three weekly doses of CRLX101 resulted in significant anti-tumor activity that was superior to irinotecan or topotecan. Most notably, complete tumor regression was observed in all animals bearing H1299 tumors and in the majority of animals with disseminated Ewing’s sarcoma, Daudi, and Karpas 299 lymphoma tumors. Additionally, CRLX101 was found to be highly effective in tumor models that respond poorly to treatment with irinotecan (MDA-MB-231, Panc-1, and HT29), suggesting CRLX101’s potential ability to overcome drug resistance in these models. These findings provide compelling support that CDP as a nanopharmaceutical technology has (a) successfully overcome CPT’s formulation, toxicity, and pharmacokinetic challenges, (b) maintained CPT in its active form resulting in potent anti-tumor activity, (c) increased intracellular CPT concentrations, (d) enhanced tumor drug localization and retention, and (e) produced a sustained therapeutic effect with superior efficacy over approved Topo 1 inhibitors.

Because CRLX101 as a nanopharmaceutical has an increased therapeutic window, an important clinical implication is that it can be more easily combined with other chemotherapeutic agents for synergistic activity. Studies were conducted with CRLX101 combined with highly efficacious chemotherapeutic agents, including cisplatin, carboplatin, paclitaxel, and gemcitabine. Antitumor activity of such drug combinations were tested in xenograft tumor mouse models, including human A2780 and SK-OV-3 ovarian carcinoma [25]. In these studies, all CRLX101 drug combinations tested were found to exhibit greater than additive efficacy, compared to the single-agent activity observed with each of the individual chemotherapeutic agents. Furthermore, in the case of cisplatin, paclitaxel, and gemcitabine, the observed enhanced efficacy from combination therapy was achieved without additive toxicity. A key clinical implication from these findings is that CRLX101 can be developed as a highly versatile chemotherapeutic agent that has significant monotherapy activity, with the potential for combination with multiple widely-used oncology agents.

## 3 CLINICAL PHASE 1 STUDY WITH CRLX101

Data from the preclinical studies of CRLX101 have confirmed that the CDP nanopharmaceutical technology has successfully conferred superior pharmacokinetics, improved tolerability, enhanced pharmacodynamics, and increased efficacy to CPT. Considering the original promise of CPT as an anti-tumor agent and the favorable pharmaceutical profile of its nanopharmaceutical design and configuration, CRLX101 is considered a promising oncology agent and clinical development was initiated. A Phase 1/2a study is currently underway and a Phase 2 study is being planned.

## 4 PERSPECTIVE

The application of polymeric nanoparticles for addressing formulation and pharmacokinetic challenges has long been a hot topic in drug delivery research and development. Advances in polymer chemistry and a better understanding of tumor microenvironment biology have supported the progress of nanopharmaceuticals as a new class of therapeutic agents. While other nanometer-scale drug carrier technologies and approaches have demonstrated an ability to overcome some of the formulation and pharmacokinetic hurdles, development of CRLX101 as a first-in-class nanopharmaceutical has established that cyclodextrin polymeric nanoparticle (CDP) technology is a highly versatile nanopharmaceutical platform technology that confers significant biological advantages to active pharmaceutical ingredients, including target-tissue localization, enhanced cellular uptake, and slow drug release kinetics resulting in sustained therapeutic drug concentrations in target cells. Together, these biological properties have a strong potential to translate therapy into significant impact on clinical outcome. 

Cerulean Pharma Inc. is advancing CRLX101 in Phase 1/2a clinical development. One of the key objectives of the study will be to investigate the activity of CRLX101 in a variety of tumor types. Another objective of the study will be to collect more long-term safety data in patients who are responding to CRLX101 and exhibiting stable disease. These findings will be critical for establishing CRLX101’s potential as a new oncology agent and for developing human proof-of-concept data for CDP-based nanopharmaceuticals.

## Figures and Tables

**Table 1:** *In Vitro* Cytotoxicity of CRLX101 and CPT Against Multiple Human Cancer Cell Lines Determined by a 48 Hour MTS Assay

Cell Line	IC_50_ CPT [µM]	IC_50_ IT-101 [µM]
NCI-H1299 NSCLC	0.100	0.320
HT-29 Colorectal	0.020	0.218
A2780 Ovarian	0.007	0.039
PANC-1 Pancreatic	0.050	0.100
DAUDI Burkitt’s Lymphoma	0.060	0.170

IC_50_: 50% inhibitory concentration. Data from T. Schluep (personal communication), references [7, 22].

**Table 2:** Biodistribution of CRLX101 in Nude Mice Bearing Subcutaneous, Human LS174T Colorectal Cancer Xenografts

Tissue \ Time Point	Level of Released, Active CPT (ng/mL or ng/g)			
	CRLX101		CPT	
	24 hrs	48 hrs	24 hrs	48 hrs
Plasma	72 ± 9	3 ± 3	0 ± 0	0 ± 0
Tumor	183 ± 115	55 ± 22	1 ± 0	0 ± 0
Liver	241 ± 132	121 ± 57	106 ± 40	108 ± 74
Spleen	45 ± 42	13 ± 17	49 ± 15	0 ± 0
Lung	57 ± 43	11 ± 9	8 ± 15	0 ± 0
Heart	23 ± 21	5 ± 5	0 ± 0	14 ± 0

Plasma and tissue collection at 24 and 48 hours after single-dose administration at respective MTDs (3 mg/kg Intraperitoneally for CPT and 24 mg/kg CPT equivalent dose intravenously for CRLX101). Data are from reference [23].

**Table 3:** Pharmacokinetic Parameters of CRLX101 and CPT in Sprague Dawley rats. Values from Reference [23]

Pharmacokinetic Parameter	CPT (1.0 mg/kg CPT)	IT-101 (0.9 mg/kg CPT Equivalents)	
		Polymer Bound CPT	Released CPT
C_max_ (µg/mL)	0.4	9.1	0.2
V_ss _(mL)	1306	47.2	n/a
CL (mL/h)	1534	3.4	n/a
Terminal half-life (h)	1.3	17.2	n/a
AUC (µg h/mL)	0.2	66.9	0.2

**Table 4:** Maximum Tolerated Dose (MTD) of CRLX101 on Various Dosing Schedules in Athymic Nude Mice [23]

Dosing Schedule	MTD (mg/kg)
qd x 1	24
5/2/5/2/5	3
q4d x 5	10
qwk x 3	15

MTD was defined as the dose level below which animals exhibited a mean body weight loss greater than 20% or greater than 10% of animal deaths in a particular group.

## Footnotes

1 Originally published data indicated a half-life of 1.7 hours in human plasma (J. Cheng, K.T. Khin, G.S. Jensen, A. Liu & M.E. Davis Bioconjug Chem 14, 1007-17. (2003)). However, subsequent repeat analysis indicated a correct half-life of 41 hours in human plasma when the pH of the human plasma was adjusted to pH 7.4. Freshly reconstituted human plasma had a pH of approx. 9.5 prior to pH adjustment.

2 Irinotecan was dosed at 100 mg/kg weekly times three (qwk x 3), CPT was dosed at 3 mg/kg every four days times five (q4d x 5), and CRLX101 was dosed daily (qd x 1) at 18.3 mg/kg, 5/2/5/2/5 at 3.2 mg/kg, q4d x 5 at 9.7 mg/kg, and qwk x 3 at 18.3 mg/kg in CPT equivalents.
